# Length of Variable Numbers of Tandem Repeats in the Carboxyl Ester Lipase (CEL) Gene May Confer Susceptibility to Alcoholic Liver Cirrhosis but Not Alcoholic Chronic Pancreatitis

**DOI:** 10.1371/journal.pone.0165567

**Published:** 2016-11-01

**Authors:** Karianne Fjeld, Sebastian Beer, Marianne Johnstone, Constantin Zimmer, Joachim Mössner, Claudia Ruffert, Mario Krehan, Christian Zapf, Pål Rasmus Njølstad, Stefan Johansson, Peter Bugert, Fabio Miyajima, Triantafillos Liloglou, Laura J. Brown, Simon A. Winn, Kelly Davies, Diane Latawiec, Bridget K. Gunson, David N. Criddle, Munir Pirmohamed, Robert Grützmann, Patrick Michl, William Greenhalf, Anders Molven, Robert Sutton, Jonas Rosendahl

**Affiliations:** 1 KG Jebsen Center for Diabetes Research, Department of Clinical Science, University of Bergen, Bergen, Norway; 2 Center for Medical Genetics and Molecular Medicine, Haukeland University Hospital, Bergen, Norway; 3 Department of Internal Medicine, Neurology and Dermatology, Division of Gastroenterology and Rheumatology, University of Leipzig, Leipzig, Germany; 4 NIHR Liverpool Pancreas Biomedical Research Unit, Royal Liverpool University Hospital, Institute of Translational Medicine, University of Liverpool, Liverpool, United Kingdom; 5 Department of Internal Medicine I, Martin Luther University, Halle, Germany; 6 Department of Pediatrics, Haukeland University Hospital, Bergen, Norway; 7 Institute of Transfusion Medicine and Immunology, Medical Faculty Mannheim, Heidelberg University, German Red Cross Blood Service of Baden-Württemberg-Hessen, Mannheim, Germany; 8 Department of Molecular and Clinical Pharmacology, Institute of Translational Medicine, University of Liverpool, Liverpool, United Kingdom; 9 Department of Molecular and Clinical Cancer Medicine, Institute of Translational Medicine, University of Liverpool, Liverpool, United Kingdom; 10 NIHR Birmingham Liver Biomedical Research Unit, Queen Elizabeth Hospital and University of Birmingham, Birmingham, United Kingdom; 11 Department of Cellular & Molecular Physiology, Institute of Translational Medicine, University of Liverpool, Liverpool, United Kingdom; 12 Department of Surgery, Friedrich-Alexander-Universität Erlangen-Nürnberg, Erlangen, Germany; 13 Gade Laboratory for Pathology, Department of Clinical Medicine, University of Bergen, Bergen, Norway; 14 Department of Pathology, Haukeland University Hospital, Bergen, Norway; University of Thessaly Faculty of Medicine, GREECE

## Abstract

**Background:**

Carboxyl-ester lipase (CEL) contributes to fatty acid ethyl ester metabolism, which is implicated in alcoholic pancreatitis. The *CEL* gene harbours a variable number of tandem repeats (VNTR) region in exon 11. Variation in this VNTR has been linked to monogenic pancreatic disease, while conflicting results were reported for chronic pancreatitis (CP). Here, we aimed to investigate a potential association of *CEL* VNTR lengths with alcoholic CP.

**Methods:**

Overall, 395 alcoholic CP patients, 218 patients with alcoholic liver cirrhosis (ALC) serving as controls with a comparable amount of alcohol consumed, and 327 healthy controls from Germany and the United Kingdom (UK) were analysed by determination of fragment lengths by capillary electrophoresis. Allele frequencies and genotypes of different VNTR categories were compared between the groups.

**Results:**

Twelve repeats were overrepresented in UK ACP patients (*P* = 0.04) compared to controls, whereas twelve repeats were enriched in German ALC compared to alcoholic CP patients (*P =* 0.03). Frequencies of *CEL* VNTR lengths of 14 and 15 repeats differed between German ALC patients and healthy controls (*P* = 0.03 and 0.008, respectively). However, in the genotype and pooled analysis of VNTR lengths no statistical significant association was depicted. Additionally, the 16–16 genotype as well as 16 repeats were more frequent in UK ALC than in alcoholic CP patients (*P* = 0.034 and 0.02, respectively). In all other calculations, including pooled German and UK data, allele frequencies and genotype distributions did not differ significantly between patients and controls or between alcoholic CP and ALC.

**Conclusions:**

We did not obtain evidence that *CEL* VNTR lengths are associated with alcoholic CP. However, our results suggest that *CEL* VNTR lengths might associate with ALC, a finding that needs to be clarified in larger cohorts.

## Introduction

Chronic pancreatitis (CP) is a recurring and painful inflammatory disease leading to exocrine and endocrine insufficiency in many patients [1,2]. In the industrialized world, alcohol abuse is the predominant cause whereas smoking seems to be an independent risk factor. In non-alcoholic CP, several genetic associations have recently been described. While variants in *PRSS1* (cationic trypsinogen), *CFTR* (cystic fibrosis transmembrane conductance regulator), *SPINK1* (serine protease, kazal type 1), *CTRC* (chymotrypsinogen C), *CPA1* (carboxypeptidase A1) and the *CLDN2*-*MORC4* locus have been identified to increase risk of CP development, a rare variant in *PRSS2* (anionic trypsinogen) and a common variant in the *PRSS1-PRSS2* locus are protective [3–10].

In alcoholic CP, genetic associations have been described for the variants p.N34S in *SPINK1*, p.R254W in *CTRC*, and common variants of the *PRSS1-PRSS2* and *CLDN2*-*MORC4* locus [6,8,11]. This is an interesting finding because only five percent of patients with alcohol abuse develop alcoholic CP, indicating that genetic or other factors contribute to disease development. As such, further genetic associations with alcoholic CP might be identified by hypothesis-driven as well as hypothesis-free approaches.

Carboxyl-ester lipase (CEL) is secreted into the pancreatic juice and contributes to the hydrolysis of dietary lipids in the duodenum [12,13]. Pancreatitis is caused by intracellular calcium overload, which can be induced by the presence of fatty acids and fatty acid ethyl esters (FAEEs) within the pancreas [14]. FAEEs are a product of the non-oxidative metabolism of ethanol and fatty acids and CEL is involved in this metabolic pathway [15–17].

A hybrid allele comprising parts of *CEL* and the neighbouring *CEL* pseudogene (*CELP*) confers disease risk in non-alcoholic and alcoholic CP [18]. A variable number of tandem repeats (VNTR), consisting of nearly identical 33-base pair segments, is located in *CEL* exon 11. This VNTR encodes a repetition of 11 amino acids located in the C-terminal part of the protein [19–21]. Variations within this region have been linked to *CEL*-MODY, an autosomal dominantly inherited disease characterized by monogenic diabetes and pancreatic exocrine dysfunction [19]. The most common *CEL* allele in the general population contains a VNTR of 16 segments, but VNTR lengths can vary between 3 and 23 repeats [19–23]. The definition of long and short VNTR repeats, however, remains ambiguous in the literature and may contribute to differing results when patients are analysed.

A Japanese study described an association of longer *CEL* VNTR repeats (designated as the *L* allele) with alcoholic CP, whereas no association was found in non-alcoholic CP patients and in patients with alcohol abuse and no signs of CP [24]. Notably, the distribution of absolute repeat numbers was not stated in that study. Moreover, this result was challenged by a recent investigation of European CP patients, in that no statistically significant association between *CEL* VNTR lengths in alcoholic and non-alcoholic CP patients was identified [25]. To clarify the role of the *CEL* VNTR in alcoholic CP, we have here investigated alcoholic CP patients as well as patients with alcoholic liver cirrhosis (ALC) and healthy controls from Germany and the United Kingdom. We employed an established screening method that accurately identifies the length of the *CEL* VNTR [25].

## Materials and Methods

### Study Subjects

The medical ethical review committee of the University Leipzig (Ethic committee of the medical faculty of the University Leipzig, Germany; http://home.uni-leipzig.de/ethik/index.htm; Approval: 376-11-12122011) and the Multicentre Research Ethics Committees for Liverpool (Approval 10/WN003/46 extended as 16/WA/0057 from Wales Research Ethics Committee 4, United Kingdom, UK; Approval 11/NW/0347 and 09/H1010/75 from North West Haydock Research Ethics Committee 5, UK) approved the study protocol and all patients gave written informed consent. Diagnosis of CP was based on two or more of the following findings: presence of a typical history of recurrent pancreatitis or recurrent abdominal pain typical for CP, pancreatic calcifications and/or pancreatic ductal irregularities revealed by endoscopic retrograde pancreatography or by magnetic resonance imaging of the pancreas and/or pathological sonographic findings. Alcoholic CP was defined in patients who had consumed more than 80 g/d alcohol for at least two years in men and more than 60 g/d for women.

ALC was diagnosed according to results of liver biopsy (fibrosis stage 4) or due to unequivocal clinical and laboratory findings in men who consumed more than 80 g/d and in women who consumed more than 60 g/d for at least 10 years. Such findings were abnormal aminotransferase level, gamma glutamyl transpeptidase level, coagulation tests, serum albumin concentration and platelet count, and/or complications related to liver cirrhosis including esophageal varices, ascites, hepatic encephalopathy and typical liver morphology in sonography or computed tomography. Other etiologies of liver cirrhosis were excluded by standard laboratory tests.

In total, we enrolled 293 German alcoholic CP patients (87.7% males, age range 18–73 years, median 43 years), 127 German ALC patients (77.2% males, age range 32–78 years, median 55 years), and 236 healthy German controls (50.9% males, age range 60–70 years, median 63 years). No samples overlapped with those formerly reported in [25]. From the UK in total 102 alcoholic CP patients (84% males, age range 20–72 years, median 43 years), 91 ALC patients (66% males, age range 34–80 years, median 57 years), and 91 healthy controls (40% males, age range 35–62 years, median 58 years) were enrolled.

### Genotyping

Genomic DNA was extracted from peripheral blood leukocytes according to a standard protocol (QIAmp DNA Blood Mini kit (Qiagen Cat No./ID 51106) or MagNA Pure isolation kit (Roche Cat# 03730964001)). For analysis of *CEL* VNTR lengths, polymerase chain reaction (PCR) followed by DNA fragment analysis was performed as described in [25]. Briefly, PCR was carried out using a forward primer binding upstream and partly into the first repeat of the VNTR region, and a NED-labelled reverse primer binding downstream of the VNTR, resulting in an amplified product covering the complete VNTR. The PCR products were diluted and fragment analysis performed on an ABI 3100 capillary sequencer (Applied Biosystems). In addition to a DNA size standard, a set of predetermined samples was used for calibration of VNTR lengths. In the resulting spectra, the size (base pairs) of the NED-labelled DNA fragments determined the total number of the *CEL* VNTR repeats.

### Statistics

The significance of the differences between VNTR frequencies was tested in affected individuals and controls by two-tailed Fisher's Exact or Pearson’s Chi-square test and *P*-values calculated using the Simple Interactive Statistical Analysis webpage (www.quantitativeskills.com/sisa/index.htm). *P*-values <0.05 were considered statistically significant.

## Results

*CEL* VNTR lengths ranged from 7 to 23 repeats in the overall study population. In each of the three cohorts, the most frequent allele contained 16 repeats (Germany/UK: alcoholic CP, 62.4%/55.6%; ALC, 58.4%/67.0%; controls, 59.7%/57.6%). The distribution of VNTR lengths in healthy controls was comparable to that of previous studies [24, 25] and all cohorts were within Hardy-Weinberg equilibrium.

During fragment analysis, some samples (Germany/UK: alcoholic CP, n = 7/3; ALC, n = 3/3; healthy controls, n = 5/2) exhibited three NED-labelled peaks instead of the expected one or two peaks [23]. The reason is that some individuals harbour a duplication variant of *CEL*, implying that they carry an extra copy of the VNTR region [18, 23]. We have included the subjects with three VNTR lengths in our analyses. Thus, when calculating the allele frequencies the total number of alleles is slightly larger than twice the number of subjects.

### Alcoholic CP vs Healthy Controls

When comparing VNTR allele frequencies between alcoholic CP patients and controls a statistically significant difference was obtained for 12 repeats in the UK cohort, with overrepresentation in patients (3.9%) versus controls (0.5%) (*P* = 0.04; odds ratio (OR) = 7.36; 95% confidence interval (CI) = 0.91–53.39), that was not seen in the German cohorts (Tables 1 and 2). Next, the VNTR lengths were pooled into three categories of short (S, 7–15 repeats), normal (N, 16 repeats), and long (L, 17–23 repeats) alleles. In this pooled analysis, the distribution in alcoholic CP patients and healthy controls did not differ significantly in either German or UK cohorts (Tables 1 and 2, lower part). To address whether distinct genotypes (SS, SN, NN, NL, LL, SL, or the presence of *CEL* duplication variants) contribute to alcoholic CP risk, genotype frequencies were calculated in both groups. Again, there was no significant difference between alcoholic CP patients and healthy controls (Tables 3 and 4).

**Table 1:** Distribution of *CEL* VNTR lengths for alcoholic chronic pancreatitis (ACP) patients and controls from Germany.

		Length frequency				
		ACP	Controls	*P*-value^a^	Odds ratio	95% CI
		(n = 293)	(n = 236)			
Number of VNTR repeats	10	0.002 (1)	0.004 (2)	0.59	0.40	0.04–4.45
	11	0.010 (6)	0.010 (5)	1.00	0.96	0.29–3.18
	12	0.002 (1)	0.010 (5)	0.09	0.16	0.02–1.41
	13	0.054 (32)	0.042 (20)	0.39	1.30	0.74–2.31
	14	0.133 (79)	0.117 (56)	0.44*	1.16	0.80–1.67
	15	0.138 (82)	0.170 (81)	0.15*	0.79	0.56–1.10
	16	0.624 (370)	0.597 (285)	0.38*	1.12	0.87–1.43
	17	0.030 (18)	0.046 (22)	0.20	0.65	0.34–1.22
	18	0.003 (2)	0.002 (1)	1.00	1.61	0.15–17.82
	21	0.002 (1)	-	-	-	-
	23	0.002 (1)	-	-	-	-
	Sum	1.000 (593)	1.000 (477)	-	-	-
Pooled VNTR lengths	Short (<16)	0.339 (201)	0.352 (168)	0.65*	0.94	0.73–1.22
	Normal (16)	0.624 (370)	0.597 (285)	0.38*	1.12	0.87–1.43
	Long (>16)	0.037 (22)	0.048 (23)	0.44	0.76	0.42–1.38
	Sum	1.000 (593)	1.000 (477)	-	-	-

^a^ *P*-value calculated by two-tailed Fisher`s exact test or Pearson’s Chi-square test (*) with one degree of freedom

**Table 2:** Distribution of *CEL* VNTR lengths for alcoholic chronic pancreatitis (ACP) patients and controls from the United Kingdom.

		Length frequency				
		ACP	Controls	*P*-value^a^	Odds ratio	95% CI
		(n = 102)	(n = 91)			
Number of VNTR repeats	7	0.001 (2)	-	-	-	-
	11	0.005 (1)	-	-	-	-
	12	0.039 (8)	0.005 (1)	0.04	7.36	0.91–59.39
	13	0.058 (12)	0.076 (14)	0.54	0.75	0.34–1.66
	14	0.140 (29)	0.158 (29)	0.67	0.87	0.50–1.52
	15	0.135 (28)	0.147 (27)	0.77	0.91	0.51–1.61
	16	0.556 (115)	0.576 (106)	0.68*	0.92	0.62–1.37
	17	0.048 (10)	0.038 (7)	0.81	1.28	0.48–3.44
	18	0.001 (2)	-	-	-	-
	Sum	1.000 (207)	1.000 (184)	-	-	-
Pooled VNTR lengths	Short (<16)	0.386 (80)	0.386 (71)	0.99*	1.00	0.67–1.51
	Normal (16)	0.556 (115)	0.576 (106)	0.68	0.92	0.62–1.37
	Long (>16)	0.058 (12)	0.038 (7)	0.48	1.56	0.60–4.04
	Sum	1.000 (207)	1.000 (184)	-	-	-

^a^ *P*-value calculated by two-tailed Fisher`s exact test or Pearson’s Chi-square test (*) with one degree of freedom

**Table 3:** Genotype distribution of *CEL* VNTR lengths for alcoholic chronic pancreatitis (ACP) patients and controls from Germany.

Genotypes of pooled lengths^a^	Frequency				
	ACP	Controls	*P*-value^b^	Odds ratio	95% CI
	(n = 293)	(n = 236)			
SS	0.096 (28)	0.123 (29)	0.33	0.75	0.44–1.31
SN	0.423 (124)	0.403 (95)	0.63*	1.09	0.77–1.54
NN	0.392 (115)	0.364 (86)	0.51*	1.13	0.79–1.61
NL	0.038 (11)	0.055 (13)	0.40	0.67	0.29–1.52
LL	-	-	-	-	-
SL	0.027 (8)	0.034(8)	0.80	0.80	0.30–2.17
Duplication alleles^c^	0.024 (7)	0.021 (5)	1.00	1.13	0.35–3.61
Sum	1.000 (293)	1.000 (236)	-	-	-

^a^ Short (S), 4–15 repeats; Normal (N), 16 repeats; Long (L), 17–23 repeats

^b^ *P*-value calculated by two-tailed Fisher`s exact test or Pearson’s Chi-square test (*) with one degree of freedom

^c^ Genotypes of duplication alleles are listed in Table 13

**Table 4:** Genotype distribution of *CEL* VNTR lengths for alcoholic chronic pancreatitis (ACP) patients and controls from the United Kingdom.

Genotypes of pooled lengths^a^	Frequency				
	ACP	Controls	*P*-value^b^	Odds ratio	95% CI
	(n = 102)	(n = 91)			
SS	0.176 (18)	0.121 (11)	0.32	1.56	0.69–3.50
SN	0.363 (37)	0.450 (41)	0.22*	0.69	0.39–1.24
NN	0.324 (33)	0.330 (30)	1.00	0.97	0.53–1.78
NL	0.088 (9)	0.033 (3)	0.14	2.84	0.74–10.83
LL	-	-	-	-	-
SL	0.020 (2)	0.044 (4)	0.42	0.44	0.08–2.43
Duplication alleles^c^	0.030 (3)	0.022 (2)	1.00	1.35	0.22–8.26
Sum	1.000 (102)	1.000 (91)	-	-	-

^a^ Short (S), 4–15 repeats; Normal (N), 16 repeats; Long (L), 17–23 repeats

^b^ *P*-value calculated by two-tailed Fisher`s exact test or Pearson’s Chi-square test (*) with one degree of freedom

^c^ Genotypes of duplication alleles are listed in Table 14

### ALC vs Healthy Controls

Comparison of ALC patients and healthy controls revealed a statistically significant overrepresentation of a VNTR length of 14 repeats in German patients (17.5%) vs. controls (11.7%) (*P* = 0.03; OR = 1.60; 95% CI = 1.04–2.44). In contrast, an underrepresentation of 15 repeats was found in German patients (9.7%) compared with controls (17.0%) (*P* = 0.008; OR = 0.53; 95% CI = 0.33–0.85) (Table 5). In the UK cohorts, VNTR length of 16 repeats was enriched in ALC patients (67.0%) vs. controls (57.6%), but the difference did not quite reach statistical significance (*P* = 0.06; OR = 1.50; 95% CI = 0.98–2.29). Calculations of pooled VNTR lengths and genotype distribution in cohorts from both countries did not show differences between the groups (Tables 5–8).

**Table 5:** Distribution of *CEL* VNTR lengths for alcoholic liver cirrhosis (ALC) patients and controls from Germany.

		Length frequency				
		ALC	Controls	*P*-value^a^	Odds ratio	95% CI
		(n = 127)	(n = 236)			
Number of VNTR repeats	9	0.004 (1)	-	-	-	-
	10	-	0.004 (2)	-	-	-
	11	0.012 (3)	0.010 (5)	1.00	1.12	0.26–4.70
	12	0.016 (4)	0.010 (5)	0.73	1.50	0.40–5.61
	13	0.066 (17)	0.042 (20)	0.16	1.62	0.83–3.15
	14	0.175 (45)	0.117 (56)	0.03*	1.60	1.04–2.44
	15	0.097 (25)	0.170 (81)	0.008	0.53	0.33–0.85
	16	0.584 (150)	0.597 (285)	0.72*	0.94	0.69–1.29
	17	0.043 (11)	0.046 (22)	1.00	0.92	0.44–1.94
	18	0.004 (1)	0.002 (1)	1.00	1.86	0.12–29.85
	Sum	1.000 (257)	1.000 (477)	-	-	-
Pooled VNTR lengths	Short (<16)	0.370 (95)	0.352 (168)	0.64*	1.08	0.79–1.48
	Normal (16)	0.584 (150)	0.597 (285)	0.72*	0.94	0.69–1.29
	Long (>16)	0.047 (12)	0.048 (23)	1.00	0.97	0.47–1.98
	Sum	1.000 (257)	1.000 (477)	-	-	-

^a^ *P*-value calculated by two-tailed Fisher`s exact test or Pearson’s Chi-square test (*) with one degree of freedom

**Table 6:** Distribution of *CEL* VNTR lengths for alcoholic liver cirrhosis (ALC) patients and controls from the United Kingdom.

		Length frequency				
		ALC	Controls	*P*-value^a^	Odds ratio	95% CI
		(n = 91)	(n = 91)			
Number of VNTR	10	0.005 (1)	-	-	-	-
	12	0.011 (2)	0.005 (1)	1.00	2.00	0.18–22.25
repeats	13	0.043 (8)	0.076 (14)	0.20	0.55	0.23–1.34
	14	0.103 (19)	0.158 (29)	0.12	0.61	0.33–1.14
	15	0.151 (28)	0.147 (27)	1.00	1.04	0.59–1.84
	16	0.670 (124)	0.576 (106)	0.06*	1.50	0.98–2.29
	17	0.016 (3)	0.038 (7)	0.22	0.42	0.11–1.64
	Sum	1.000 (185)	1.000 (184)	-	-	-
Pooled VNTR lengths	Short (<16)	0.314 (58)	0.386 (71)	0.15*	0.73	0.47–1.12
	Normal (16)	0.670 (124)	0.576 (106)	0.06*	1.50	0.98–2.29
	Long (>16)	0.047 (3)	0.038 (7)	0.22	0.42	0.11–1.64
	Sum	1.000 (185)	1.000 (184)	-	-	-

^a^ *P*-value calculated by two-tailed Fisher`s exact test or Pearson’s Chi-square test (*) with one degree of freedom

**Table 7:** Genotype distribution of *CEL* VNTR lengths for alcoholic liver cirrhosis (ALC) patients and controls from Germany.

Genotypes of pooled lengths^a^	Frequency				
	ALC	Controls	*P*-value^b^	Odds ratio	95% CI
	(n = 127)	(n = 236)			
SS	0.134 (17)	0.123 (29)	0.74	1.10	0.58–2.10
SN	0.425 (54)	0.403 (95)	0.68*	1.10	0.71–1.70
NN	0.354 (45)	0.364 (86)	0.85*	0.96	0.61–1.50
NL	0.047 (6)	0.055 (13)	0.81	0.85	0.32–2.30
LL	0.008 (1)	-	-	-	-
SL	0.008 (1)	0.034 (8)	0.17	0.23	0.03–1.83
Duplication alleles^c^	0.024 (3)	0.021 (5)	1.00	1.12	0.26–4.76
Sum	1.000 (127)	1.000 (236)	-	-	-

^a^ Short (S), 4–15 repeats; Normal (N), 16 repeats; Long (L), 17–23 repeats

^b^ *P*-value calculated by two-tailed Fisher`s exact test or Pearson’s Chi-square test (*) with one degree of freedom

^c^ Genotypes of duplication alleles are listed in Table 13

**Table 8:** Genotype distribution of *CEL* VNTR lengths for alcoholic liver cirrhosis (ALC) patients and controls from the United Kingdom.

Genotypes of pooled lengths^a^	Frequency				
	ALC	Controls	*P*-value^b^	Odds ratio	95% CI
	(n = 91)	(n = 91)			
SS	0.088 (8)	0.121 (11)	0.48	0.70	0.27–1.83
SN	0.385 (35)	0.450 (41)	0.37*	0.76	0.42–1.38
NN	0.473 (43)	0.330 (30)	0.07	1.82	1.00–3.32
NL	-	0.033 (3)	-	-	-
LL	-	-	-	-	-
SL	0.022 (2)	0.044 (4)	0.68	0.49	0.09–2.74
Duplication alleles^c^	0.033 (3)	0.022 (2)	1.00	1.52	0.25–9.30
Sum	1.000 (91)	1.000 (91)	-	-	-

^a^ Short (S), 4–15 repeats; Normal (N), 16 repeats; Long (L), 17–23 repeats

^b^ *P*-value calculated by two-tailed Fisher`s exact test or Pearson’s Chi-square test (*) with one degree of freedom

^c^ Genotypes of duplication alleles are listed in Table 14

### Alcoholic CP vs ALC

To examine a potential influence of alcohol consumption, alcoholic CP and ALC patients were compared. Here, in the German cohorts, the VNTR lengths differed for 12 repeats (0.2% in alcoholic CP vs. 1.6% in ALC patients; *P* = 0.03; OR = 0.11; 95% CI = 0.01–0.96) (Table 9). Analyses of the pooled VNTR lengths and genotypes in the German cohorts did not identify any associations. In the UK cohorts, VNTR lengths differed significantly for 16 repeats between alcoholic CP (55.6%) and ALC patients (67.0) (*P* = 0.02; OR = 0.62; 95% CI = 0.41–0.93) (Tables 9–11). Genotype data also revealed an overrepresentation of the NN genotype in ALC patients (47.3%) compared to alcoholic CP patients (32.4%) (*P* = 0.034; OR = 0.53; 95% CI = 0.30–0.96) (Table 12).

**Table 9:** Distribution of *CEL* VNTR lengths for alcoholic chronic pancreatitis (ACP) and alcoholic liver cirrhosis (ALC) patients from Germany.

		Length frequency				
		ACP	ALC	*P*-value^a^	Odds ratio	95% CI
		(n = 293)	(n = 127)			
Number of VNTR repeats	9	-	0.004 (1)	-	-	-
	10	0.002 (1)	-	-	-	-
	11	0.010 (6)	0.012 (3)	1.00	0.86	0.22–3.49
	12	0.002 (1)	0.016 (4)	0.03	0.11	0.01–0.96
	13	0.054 (32)	0.066 (17)	0.52	0.81	0.44–1.48
	14	0.133 (79)	0.175 (45)	0.11*	0.72	0.49–1.08
	15	0.138 (82)	0.097 (25)	0.11	1.49	0.93–2.39
	16	0.624 (370)	0.584 (150)	0.27*	1.18	0.88–1.60
	17	0.030 (18)	0.043 (11)	0.41	0.70	0.33–1.50
	18	0.003 (2)	0.004 (1)	1.00	0.87	0.08–9.60
	21	0.002 (1)	-	-	-	-
	23	0.002 (1)	-	-	-	-
	Sum	1.000 (593)	1.000 (257)	-	-	-
Pooled VNTR lengths	Short (<16)	0.339 (201)	0.370 (95)	0.39*	0.87	0.64–1.19
	Normal (16)	0.624 (370)	0.584 (150)	0.27*	1.18	0.88–1.60
	Long (>16)	0.037 (22)	0.047 (12)	0.57	0.79	0.38–1.62
	Sum	1.000 (593)	1.000 (257)	-	-	-

^a^ *P*-value calculated by two-tailed Fisher`s exact test or Pearson’s Chi-square test (*) with one degree of freedom

**Table 10:** Distribution of *CEL* VNTR lengths for alcoholic chronic pancreatitis (ACP) and alcoholic liver cirrhosis (ALC) patients from the United Kingdom.

		Length frequency				
		ACP	ALC	*P*-value^a^	Odds ratio	95% CI
		(n = 102)	(n = 91)			
Number of VNTR repeats	7	0.001 (2)	-	-	-	-
	10	-	0.005 (1)	-	-	-
	11	0.005 (1)	-	-	-	-
	12	0.039 (8)	0.011 (2)	0.11	3.68	0.77–17.55
	13	0.058 (12)	0.043 (8)	0.65	1.36	0.54–3.48
	14	0.140 (29)	0.103 (19)	0.28	1.42	0.77–2.64
	15	0.135 (28)	0.151 (28)	0.67	0.88	0.50–1.54
	16	0.556 (115)	0.670 (124)	0.02*	0.62	0.41–0.93
	17	0.048 (10)	0.016 (3)	0.09	3.08	0.83–11.37
	18	0.001 (2)	-	-	-	-
	Sum	1.000 (207)	1.000 (185)	-	-	-
Pooled VNTR lengths	Short (<16)	0.386 (80)	0.314 (58)	0.13*	1.38	0.91–2.10
	Normal (16)	0.556 (115)	0.670 (124)	0.02*	0.62	0.41–0.93
	Long (>16)	0.058 (12)	0.016 (3)	0.04	3.73	1.04–13.44
	Sum	1.000 (207)	1.000 (185)	-	-	-

^a^ *P*-value calculated by two-tailed Fisher`s exact test or Pearson’s Chi-square test (*) with one degree of freedom

**Table 11:** Genotype distribution of *CEL* VNTR lengths for alcoholic chronic pancreatitis (ACP) and alcoholic liver cirrhosis (ALC) patients from Germany.

Genotypes of pooled lengths^a^	Frequency				
	ACP	ALC	*P*-value^b^	Odds ratio	95% CI
	(n = 293)	(n = 127)			
SS	0.096 (28)	0.134 (17)	0.30	0.68	0.36–1.30
SN	0.423 (124)	0.425 (54)	0.97*	0.99	0.65–1.51
NN	0.392 (115)	0.354 (45)	0.46*	1.18	0.76–1.82
NL	0.038 (11)	0.047 (6)	0.60	0.79	0.28–2.18
LL	-	0.008 (1)	-	-	-
SL	0.027 (8)	0.008 (1)	0.29	3.54	0.44–28.58
Duplication alleles^c^	0.024 (7)	0.024 (3)	1.00	1.01	0.26–3.98
Sum	1.000 (293)	1.000 (127)	-	-	-

^a^ Short (S), 4–15 repeats; Normal (N), 16 repeats; Long (L), 17–23 repeats

^b^ *P*-value calculated by two-tailed Fisher`s exact test or Pearson’s Chi-square test (*) with one degree of freedom

^c^ Genotypes of duplication alleles are listed in Table 13

**Table 12:** Genotype distribution of *CEL* VNTR lengths for alcoholic chronic pancreatitis (ACP) and alcoholic liver cirrhosis (ALC) patients from the United Kingdom.

Genotypes of pooled lengths^a^	Frequency				
	ACP	ALC	*P*-value^b^	Odds ratio	95% CI	
	(n = 102)	(n = 91)				
SS	0.176 (18)	0.088 (8)	0.09	2.22	0.92–5.39
SN	0.363 (37)	0.385 (35)	0.75*	0.91	0.51–1.63
NN	0.324 (33)	0.473 (43)	0.034*	0.53	0.30–0.96
NL	0.088 (9)	-	-	-	-
LL	-	-	-	-	-
SL	0.020 (2)	0.022 (2)	1.0	0.89	0.12–6.45
Duplication alleles^c^	0.030 (3)	0.033 (3)	1.0	0.89	0.18–4.52
Sum	1.000 (102)	1.000 (91)	-	-	-

^a^ Short (S), 4–15 repeats; Normal (N), 16 repeats; Long (L), 17–23 repeats

^b^ *P*-value calculated by two-tailed Fisher`s exact test or Pearsons Chi-square test (*) with one degree of freedom

^c^ Genotypes of duplication alleles are listed in Table 14

## Discussion

Taken together, this study of patients with alcoholic CP or ALC and healthy controls from two countries has identified no evidence that *CEL* VNTR lengths are associated with alcoholic CP. This is in contrast to the previous Japanese study, which reported significant underrepresentation of the NN and overrepresentation of the NL genotype in alcoholic CP patients [24]. Of note, the method applied to analyse the number of repeats was different from ours and allele frequencies of exact VNTR lengths were not listed. Thus, we assumed that the 16-repeat allele in the present analysis corresponds to the *N*-allele, less than 16 repeats to the *S*-allele, and more than 16 repeats to the *L*-allele of the Japanese study [24]. If so, our results do not confirm the observation made in the Japanese population, but are more in line with the recent European report [25].

Explanations for this discrepancy might be methodological in that alleles have been characterised differently. In addition, there could be differences in alcohol consumption between Japanese and European populations, since the Japanese VNTR study did not include the quantity of alcohol consumed in the definition of alcoholic CP. Furthermore, a proportion of the Japanese alcoholic controls may have had ALC or alcoholic CP. This is due to the fact, that these individuals had documented attacks of acute pancreatitis, but a normal pancreatogram on endoscopic retrograde cholangiopancreaticography (ERCP), which makes alcoholic CP unlikely, but does not rule this out [26]. Moreover, there were no documented investigations to rule out ALC. Otherwise, since the number of Japanese alcoholic CP patients was rather small it is difficult to draw valid conclusions from the reported data and it would be helpful to see replication in another Japanese cohort.

Alcoholic CP and ALC can be found concomitantly in patients, indicating that common pathways for disease development might be involved. It has been reported that clinical alcoholic CP and ALC occur concurrently in around 16–20% of alcoholic CP/ALC cases, but the prevalence may be higher at a histological level [27–29]. It is generally agreed, however, that there is some unknown factor(s) that predispose individuals to one or the other disease process [30, 31]. Nevertheless, the two groups can serve as controls for each other to adjust for the influence of alcohol intake, since they share a similar amount of consumed alcohol. Therefore, a joint analysis of alcoholic CP and ALC patients is valuable when attempting to identify genetic risk factors underlying alcohol-related diseases.

In our comparison of German ALC patients with healthy controls, 14 repeats were enriched in patients while 15 repeats were underrepresented. Further, 12 repeats were enriched in German ALC compared to alcoholic CP patients. Additionally, 16 repeats were overrepresented in ALC patients from the UK in comparison to alcoholic CP patients and with borderline significance in comparison to controls. As well the NN genotype was more frequent in ALC than in alcoholic CP patients. Thus, these results indicate a possible association of *CEL* VNTR lengths with the disease pathogenesis of ALC, however, the definite repeat lengths cannot be defined in this stage. A possible pathophysiological explanation for how *CEL* VNTR length contributes to ALC development might be the determination of plasma lipid levels, as *CEL* VNTR length seems to influence total and low density lipoprotein cholesterol levels [32]. This would be in line with findings in a recent genome wide associations study that identified three risk loci for ALC involved in lipid metabolic processes [33]. Further studies are warranted with larger ALC cohorts to determine whether our findings can be attributed to a population bias from one or more genes, which our data suggest is more likely, or to a specific high-risk allele.

In this study we used blood donors as controls, which might bias the results since some controls may suffer from alcoholic CP or ALC. Still, this potential confounder is surmised to be negligible due to the low prevalence of alcoholic CP and ALC in the general population as well as the strict screening protocols that blood donors have to undergo. In addition, allele frequencies and genotype data in our control group are comparable to former reports, and as such indicate that the selected control group is suitable for the conclusions drawn. Another limitation of this study could be that our method cannot distinguish samples carrying an extra copy of the *CEL* VNTR (e.g. with 14-16-16 repeats) from those samples that are truly heterozygous with regard to VNTR length (e.g. with 14–16 repeats). Consequently, we might have underestimated the number of carriers with three copies of the VNTR. Nevertheless from our data (Tables 13 and 14) it seems reasonable to propose that carriers with three copies are similarly distributed in patients and controls. Moreover, a reanalysis of our data with samples containing three VNTR lengths excluded did not alter the results (data not shown).

**Table 13:** Genotypes of observed *CEL* duplication alleles from Germany.

Materials	Number of VNTR repeats			S/N/L^a^
Alcoholic	13	14	16	SSN
chronic	14	15	16	SSN
pancreatitis	14	15	16	SSN
(n = 7)	14	15	16	SSN
	14	16	17	SNL
	14	16	17	SNL
	14	16	23	SNL
Alcoholic liver	14	15	16	SSN
cirrhosis	14	15	17	SSL
(n = 3)	15	16	17	SNL
Controls	11	15	16	SSN
(n = 5)	13	14	16	SSN
	14	15	16	SSN
	14	16	17	SNL
	15	16	17	SNL

^a^ Short (S), 4–15 repeats; Normal (N), 16 repeats; Long (L), 17–23 repeats

**Table 14:** Genotypes of observed *CEL* duplication alleles from the United Kingdom.

Materials	Number of VNTR repeats S/N/L^a^			
Alcoholic				
chronic	14	15	16	SSN
pancreatitis	14	15	16	SSN
(n = 3)	15	16	18	SNL
Alcoholic liver	14	15	16	SSN
cirrhosis	14	15	16	SSN
(n = 3)	14	16	17	SNL
Controls	13	14	16	SSN
(n = 2)	14	15	16	SSN

^a^ Short (S), 4–15 repeats; Normal (N), 16 repeats; Long (L), 17–23 repeats

In conclusion, we present a comprehensive analysis of *CEL* VNTR lengths in cohorts of patients with alcoholic CP or ALC and healthy controls from Germany and the UK. In line with the former European study [25], we did not identify any association between VNTR lengths allele frequencies or genotypes and alcoholic CP. Further work needs to be undertaken to determine how *CEL* variants may affect the development and progression of ALC.
